# The relationship between activities of daily living and speech impediments based on evidence from statistical and machine learning analyses

**DOI:** 10.3389/fpubh.2025.1491527

**Published:** 2025-02-06

**Authors:** Liu Jun, Hongguo Li, Yu Mao, Lan Hu, Dan Wu

**Affiliations:** Traditional Chinese Medicine Department, The Fourth Hospital of Changsha, Changsha, Hunan, China

**Keywords:** activities of daily living, speech impediments, machine learning algorithm, the China health and retirement longitudinal survey, Barthel index, support vector machine, decision tree, logistic regression

## Abstract

**Introduction:**

Speech impediments (SIs) are increasingly prevalent among middle-aged and older adults, raising concerns within public health. Early detection of potential SI in this demographic is critical. This study investigates the potential of Activities of Daily Living (ADL) as a predictive marker for SI, utilizing data from the 2018 China Health and Retirement Longitudinal Study (CHARLS), which includes 10,136 individuals aged 45 and above. The Barthel Index (BI) was used to assess ADL, and the correlation between ADL and SI was examined through statistical analyses. Machine learning algorithms (Support Vector Machine, Decision Tree, and Logistic Regression) were employed to validate the findings and elucidate the underlying relationship between ADL and SI.

**Background:**

SI poses significant challenges to the health and quality of life of middle-aged and older adults, increasing the demands on community-based and home care services. In the context of global aging, it is crucial to investigate the factors contributing to SI. While the role of ADL as a potential biomarker for SI remains unclear, this study aims to provide new evidence supporting ADL as an early predictor of SI through statistical analysis and machine learning validation.

**Methods:**

Data were derived from the 2018 CHARLS national baseline survey, comprising 10,136 participants aged 45 and above. ADL was evaluated using the BI, and SI was assessed based on the CHARLS records of “Speech impediments.” Statistical analyses, including independent sample t-tests, chi-square tests, Pearson and Spearman correlation tests, and hierarchical multiple linear regression, were conducted using SPSS 25.0. Machine learning algorithms, specifically Support Vector Machine (SVM), Decision Tree (DT), and Logistic Regression (LR), were implemented in Python 3.10.2.

**Results:**

Analysis of demographic characteristics revealed that the average BI score in the “With Speech impediments” group was 49.46, significantly lower than the average score of 85.11 in the “Without Speech impediments” group. Pearson correlation analysis indicated a significant negative correlation between ADL and SI (*r* = −0.205, *p* < 0.001). Hierarchical multiple linear regression confirmed the robustness of this negative correlation across three models (B = −0.001, *β* = −0.168, *t* = −16.16, 95% CI = −0.001 to −0.001, *p* = 0.000). Machine learning algorithms validated the statistical findings, confirming the predictive accuracy of ADL for SI, with the area under the curve (AUC) scores of SVM-AUC = 0.648, DT-AUC = 0.931, and LR-AUC = 0.666. The inclusion of BI in the models improved the overall predictive performance, highlighting its positive impact on SI prediction.

**Conclusion:**

The study employed various statistical methodologies to demonstrate a significant negative correlation between ADL and SI, a finding further corroborated by machine learning algorithms. Impairment in ADL increases the likelihood of SI occurrence, underscoring the importance of maintaining ADL in middle-aged and older populations to mitigate the risk of SI.

## 1 Introduction

With the intensifying global trend of population aging, the health issues of middle-aged and older adult populations have increasingly become a central concern in public health. It is projected that from 2015 to 2050, the population aged 60 and above in developing countries will increase from 900 million to 2 billion (1). This demographic shift poses significant challenges to global healthcare systems and social security frameworks. Speech impediments (SIs) is prevalent among older populations, presenting in various forms such as fluency disorders, voice disorders, and articulation disorders. These conditions may begin in childhood and persist into adulthood, or they may arise following medical events such as trauma, stroke, muscular dystrophy, or Parkinson’s disease (2). SI not only impairs daily communication abilities in older adults but also exacerbates social isolation and psychological health problems, significantly reducing their quality of life and level of social engagement (3).

Activities of Daily Living (ADL) are crucial indicators for assessing the health status of older adults (4). ADLs encompass a range of self-care tasks essential for independent living. In this study, we utilized the Barthel model, which evaluates 10 categories of ADLs: feeding, dressing, bathing, grooming, toilet use, mobility, transfers (e.g., from bed to chair), stair climbing, bowel control, and bladder control. This comprehensive approach ensures a detailed assessment of an individual’s functional capabilities (5). Impairments in ADL are closely associated with cognitive decline, depression, and deterioration in social functioning (6, 7). Previous studies have suggested a potential association between ADL impairment and SI. SI can lead to limitations in an individual’s ability to understand and express language, thereby affecting functional communication within ADLs (8). For instance, patients with Parkinson’s disease often experience multiple functional deficits, with a progressive decline in their ability to perform ADLs. SI exacerbates these deficits, as patients are unable to use language to compensate for other cognitive and motor impairments (9, 10). In cases of primary progressive aphasia (PPA), the decline in ADL performance varies across different PPA variants over time. The impairment in ADL function may precede or coincide with the deterioration of speech function, suggesting that ADL impairment could be a potential precursor to the development of speech disorders (11). ADL impairment is often accompanied by psychological conditions such as anxiety or depression. Emotional tension arising from these psychological factors may inhibit normal speech expression, or the limitation of ADL may reduce opportunities for social interaction, both of which can exacerbate SI (12). Studies on voice disorders have shown that a decline in quality of life directly affects speech activity and participation restrictions. ADL impairment can influence an individual’s performance in daily speech activities, leading to further deterioration of speech function (13).

Previous research has indicated a potential link between ADL and SI. This study aims to investigate the association between ADL and SI. Utilizing baseline data from the 2018 China Health and Retirement Longitudinal Study (CHARLS), we analyzed individuals aged 45 and above to evaluate the predictive capability of ADL for SI. By integrating statistical methods with machine learning algorithms, we aim to uncover potential associations between ADL and SI, providing new scientific evidence for the early detection and intervention of SI.

## 2 Methods

### 2.1 Study population

This study utilized data from the baseline dataset of the China Health and Retirement Longitudinal Study (CHARLS), managed by the National School of Development at Peking University.^1^ CHARLS offers a comprehensive open-access database that provides detailed information on individuals, families, health status, and socio-economic factors, including categories such as “Health Status and Functioning,” “Cognition,” “Work Retirement,” and “Family Information.” The study population consisted of individuals aged 45 and older, randomly selected from 150 counties or districts and 450 villages or urban areas across 28 provinces, representing the middle-aged and older adult demographic in China (14). For this analysis, data from the 2018 CHARLS wave were employed, initially including a total of 20,813 participants. Individuals under the age of 45, as well as those lacking sufficient demographic or health information, were excluded, resulting in a final analytical sample of 10,136 participants.

### 2.2 Assessment of speech impediments

The evaluation of speech impediments was based on data from the “Health Status and Functioning” section of CHARLS, specifically the “Speech impediments” variable. Participants were categorized into two groups: ‘With Speech impediments’ and ‘Without Speech impediments’.

### 2.3 Assessment of ADL

The Barthel Index (BI) was employed as the instrument for assessing ADL in this study. The BI is a well-established tool that evaluates an individual’s capability to perform 10 basic daily activities independently: “Feeding,” “Bathing,” “Grooming,” “Dressing,” “Bowel Control,” “Bladder Control,” “Toilet Use,” “Transfers,” “Mobility,” and “Stairs.” Each activity is scored, as shown in Table 1, reflecting the individual’s proficiency and independence in completing the task. The overall BI score, ranging from 0 to 100, is derived by summing the scores of each element. Higher BI scores indicate greater independence and proficiency in daily living activities (15–17).

**Table 1:** Activities of daily living scale.

Activities of daily living	Independent	Partially independent	Moderately dependent	Totally dependent
Mobility	15	10	5	0
Stairs	10	5	0	0
Transfers	15	10	5	0
Feeding	10	5	0	0
Bathing	5	0	0	0
Grooming	5	0	0	0
Dressing	10	5	0	0
Toilet Use	10	5	0	0
Bowel Control	10	5	0	0
Bladder Control	10	5	0	0

### 2.4 Assessment of covariates

The covariates in this study were extracted from the CHARLS dataset and included a range of demographic, behavioral, and health-related variables. These variables encompassed age, gender (male or female), residence type (Central of City/Town, Urban–Rural Integration Zone, Rural, Special Zone), education level (Primary or below, Secondary school, College or above), smoking status (Still Smoke, Used to Smoke, Never Smoked), and alcohol consumption (More than once a month, Less than once a month, None). Additionally, the dataset included information on medical conditions such as Brain Damage/Intellectual disability, Hearing Problems, and diagnoses of Hypertension, Dyslipidemia, Diabetes, Cancer, Stroke, or Memory-Related Diseases, as confirmed by a physician.

For age categorization, individuals aged between 45 and 59 years were classified as middle-aged, while those aged 60 years and above were categorized as older adult. Hypertension was defined in accordance with the 2010 Chinese Hypertension Guidelines: an average systolic blood pressure of ≥140 mmHg, an average diastolic blood pressure of ≥90 mmHg, or self-reported use of antihypertensive medications in the past 2 weeks (18). Diabetes was identified based on a fasting blood glucose level of ≥7.0 mmol/L or current use of antidiabetic medications. The criteria for dyslipidemia, as per the 2016 Chinese Adult Dyslipidemia Guidelines, included a total cholesterol level of ≥240 mg/dL, high-density lipoprotein cholesterol level of <40 mg/dL, low-density lipoprotein cholesterol level of >160 mg/dL, or a triglyceride level of ≥200 mg/dL (19). Other comorbid conditions, including cancer, stroke, and memory-related diseases, were documented based on self-reported diagnoses recorded in the CHARLS dataset, specifically under “Hearing Problem” and “Diagnosed with Cancer/Stroke/Memory-Related Disease by a Doctor.”

### 2.5 Statistical analysis

In the analysis of demographic characteristics, continuous variables such as age and BI were reported as means with standard errors (SE), while categorical variables including gender, residence, and education were presented as frequencies and percentages. To compare demographic characteristics, independent sample t-tests were performed for continuous variables, and chi-square tests were used for categorical variables to identify any significant differences. Correlation analyses were conducted using Pearson’s correlation test or Spearman’s rank correlation, depending on the distribution, to explore associations between independent variables, covariates, and dependent variables.

A comprehensive evaluation of the association between ADL and SIs was carried out using hierarchical multiple linear regression analysis. This regression analysis was performed in three stages, with each stage incorporating distinct models that included different sets of variables. Dummy variables were used as reference categories within each model. Model 1 included three primary variables: age, gender, and ADL. Model 2 extended Model 1 by adding lifestyle risk factors such as smoking status. Model 3 further built upon Model 2 by incorporating additional covariates, including Brain Damage/Intellectual disability, Hearing Problems, Stroke, Memory-Related Diseases, Hypertension, and Dyslipidemia, resulting in a total of 10 variables in the final model.

### 2.6 Machine learning algorithm

#### 2.6.1 Parameter configuration

The machine learning algorithms were developed and executed using PyCharm 2023.1.2 as the integrated development environment (IDE) on a system running Windows 11. The software development and data analysis were carried out within the Anaconda environment, employing Python version 3.10.2. To guarantee reproducibility and reliability, a random seed of 42 was consistently applied throughout the process. The original dataset was divided into training and testing subsets, with 70% allocated to the training set and 30% to the testing set. To ensure a fair and accurate evaluation of the model’s performance, the data was randomized prior to splitting. In addition, k-fold cross-validation was employed during the training phase to optimize model performance and ensure robust validation. This iterative process provided a comprehensive assessment by systematically using different portions of the data for training and validation. The final model’s effectiveness was then evaluated based on its classification performance on the testing dataset.

#### 2.6.2 Experimental models

##### 2.6.2.1 Support vector machine

SVM is a prominent supervised learning algorithm in machine learning, categorized as a generalized linear classifier. Its primary objective is to classify data points by identifying the optimal hyperplane that maximizes the margin between different classes, thereby serving as the decision boundary for the training data. A key feature of SVM is its ability to handle nonlinear classification problems effectively through the use of kernel functions, which enable it to operate in higher-dimensional spaces. In this study, we employed the radial basis function (RBF) kernel, known for its effectiveness in capturing complex, nonlinear relationships between features. To ensure optimal performance, we fine-tuned the hyperparameters, including the penalty parameter (C) and kernel coefficient (*γ*), using grid search and k-fold cross-validation. This kernel-based approach allows SVM to achieve high accuracy, particularly for high-dimensional datasets with intricate feature interactions (20, 21).

##### 2.6.2.2 Decision tree

DT is a versatile supervised learning method employed in statistics, data mining, and machine learning, capable of handling both classification and regression tasks. In classification tasks, DT are structured as hierarchical models where the leaves represent class labels and branches represent conjunctions of features that lead to these class labels. For regression, DT models predict continuous target variables, typically real numbers. DT can manage various types of data, including categorical and sequential data, enhancing their applicability across multiple analytical domains. Their inherent simplicity and interpretability make them valuable for transparent decision-making processes, particularly in decision analysis and data mining, where they provide insightful, descriptive analysis and facilitate decision-making (22–24).

##### 2.6.2.3 Logistic regression

LR is a well-established supervised learning technique in machine learning, distinct from linear regression, as it primarily addresses classification tasks, including multi-class scenarios. During training, the LR model learns from a dataset composed of multiple groups, referred to as the training set, capturing patterns essential for making classification decisions. Once trained, the model applies this learned knowledge to classify new data points within a test set, which is characterized by various features relevant to the decision-making process. The versatility and robustness of logistic regression make it a fundamental tool in supervised learning, extensively applied in scientific and medical research to tackle diverse classification problems (25–27).

#### 2.6.3 Evaluation metrics

In this study, the performance of the machine learning models was assessed using key evaluation metrics: accuracy, precision, recall, and F1-score (28, 29). Accuracy represents the ratio of correctly predicted instances to the total number of instances, providing a general measure of the model’s overall performance (Equation 1). Precision quantifies the proportion of true positive results out of all predicted positive cases, highlighting the model’s ability to avoid false positives (Equation 2). Recall (or sensitivity) measures the proportion of true positive results among all actual positive cases, reflecting the model’s ability to identify all relevant cases (Equation 3). The F1-score is the harmonic mean of precision and recall, offering a balanced assessment of a model’s accuracy, particularly in scenarios with class imbalance. A higher F1-score indicates superior model performance, as it accounts for both precision and recall (Equation 4). By employing these evaluation metrics, the study ensures a comprehensive analysis of the models’ predictive capabilities and robustness.


(1) $$\mathrm{Accuracy}=\frac{TP+TN}{TP+FP+TN+FN}$$

Formula Accuracy.


(2) $$\mathrm{Prec}i\mathrm{sion}=\frac{TP}{TP+FP}$$

Formula Precision.


(3) $$\mathrm{Recall}=\frac{TP}{TP+FN}$$

Formula Recall.


(4) $$F1=\frac{2TP}{2TP+FN+FP}=\frac{2*\mathrm{Precision}*\mathrm{Recall}}{\mathrm{Precision}+\mathrm{Recall}}$$

Formula F1-score (TP = True Positive, FP = False Positive, FN = False Negative, TN = True Negative).

## 3 Results

### 3.1 Characteristics of samples

The study excluded participants who did not meet the inclusion criteria, specifically those aged 45 years or younger and those lacking comprehensive demographic or health data (Table 2). A total of 10,136 eligible participants were included in the final analysis, of whom 10,025 (98.90%) did not have SIs, while 111 individuals (1.10%) were diagnosed with SIs. A comparative analysis of ADL, as measured by the BI, revealed that individuals with SIs had a significantly lower mean score of 49.46, compared to a mean score of 85.11 for those without SIs. This difference was statistically significant (*p* < 0.001, *t* = −10.687).

**Table 2:** Demographic characteristics of middle-aged and older adult Chinese with and without speech impediments.

Variables	Without speech impediments	With speech impediments	t/t’/χ^2^	*p*-value
No. subjects (%)	10,025(98.90%)	111(1.1%)		
Age, year			3.438	<0.001
	64.87	68.57		
SE	0.01	1.07		
Gender, n (%)			14.049	<0.001
Male	3,937(39.27%)	63(56.76%)		
Female	6,088(60.73%)	48(43.24%)		
Residence, n (%)			1.388	0.708
Central of City/Town	1,679(16.75%)	15(13.51%)		
Urban–Rural Integration Zone	666(6.64%)	9(8.11%)		
Rural	7,654(76.35%)	87(78.38%)		
Special Zone	26(0.26%)	0(0.00%)		
Education, n (%)			1.525	0.467
Primary or below	7,482(74.63%)	83(74.77%)		
Secondary school (Middle school+High school)	2,411(24.05%)	28(25.23%)		
College or above	132(1.32%)	0(0.00%)		
Smoking status, n (%)			25.648	<0.001
Still Smoke	2,194(21.89%)	23(20.72%)		
Used to Smoke	1,464(14.60%)	35(31.53%)		
Never Smoked	6,367(63.51%)	53(47.75%)		
Drinking status, n (%)				
Drink more than once a month	1954(19.49%)	20(18.02%)	0.215	0.898
Drink but less than once a month	652(6.50%)	8(7.21%)		
None	7,419(74.01%)	83(74.77%)		
Brain Damage/Intellectual disability, n (%)			276.86	<0.001
Yes	432(4.31%)	42(37.84%)		
No	9,593(95.69%)	69(62.16%)		
Hearing Problem, n (%)			85.926	<0.001
Yes	767(7.65%)	35(31.53%)		
No	9,258(92.35%)	76(68.47%)		
Stroke, n (%)				
Yes	693(6.91%)	47(42.34%)	203.626	<0.001
No	9,332(93.09%)	64(57.66%)		
Cancer, n (%)				
Yes	196(1.96%)	5(4.50%)	3.671	0.055
No	9,829(98.04%)	106(95.50%)		
Memory-Related Disease, n (%)			66.397	<0.001
Yes	348(3.47%)	20(18.02%)		
No	9,677(96.53%)	91(81.98%)		
Hypertension, n (%)			11.203	0.001
Yes	1,688(16.84%)	32(28.83%)		
No	8,337(83.16%)	79(71.17%)		
Diabetes, n (%)			2.8	0.094
Yes	680(6.78%)	12(10.81%)		
No	9,345(93.22%)	99(89.19%)		
Dyslipidemia, n (%)			4.561	0.033
Yes	1,299(12.96%)	22(19.82%)		
No	8,726(87.04%)	89(80.18%)		
Activity of daily living (The Barthel Index)			−10.687	<0.001
	85.11	49.46		
SE	0.17	3.33		

Demographic analysis demonstrated that the occurrence of SIs is significantly associated with several health and demographic factors. The mean age of participants with SIs was 68.57 years, notably higher than that of participants without SIs, whose mean age was 64.87 years, suggesting that advancing age may increase the risk of developing SIs (*p* < 0.001). Furthermore, gender-based analysis indicated that the prevalence of SIs was significantly higher in males (56.76%) compared to females (39.27%), suggesting that sex might be a critical factor influencing the development of SIs (*p* < 0.001). However, no significant associations were observed between SIs and place of residence (*p* = 0.708) or educational attainment (*p* = 0.467).

Analysis of smoking status revealed that the prevalence of SIs was significantly higher among former smokers (31.53%) compared to those who had never smoked (14.60%), indicating that a history of smoking may adversely affect speech function (*p* < 0.001). In contrast, drinking status was not significantly associated with SIs (*p* = 0.898).

A strong positive correlation was observed between SIs and both brain damage/intellectual disability; the prevalence of SIs among individuals with brain damage or intellectual disability was 37.84%, substantially higher than among those without such conditions (4.31%, *p* < 0.001). Hearing problems also emerged as a significant factor; individuals with hearing problems were more likely to experience SIs (31.53%) compared to those without hearing problems (7.65%, *p* < 0.001). The prevalence of SIs was markedly higher among stroke survivors (42.34%) than among those without a history of stroke (6.91%, *p* < 0.001), suggesting that stroke may significantly increase the risk of SIs. Similarly, individuals with memory-related diseases had a significantly higher prevalence of SIs (18.02%) compared to those without memory-related diseases (3.47%, *p* < 0.001). Regarding chronic conditions, the prevalence of SIs was significantly higher among individuals with hypertension (28.83%) compared to those without hypertension (16.84%, *p* = 0.001). Additionally, individuals with dyslipidemia showed an elevated risk of SIs (19.82%) compared to those without dyslipidemia (12.96%, *p* = 0.033). However, no significant associations were found between SIs and either diabetes (*p* = 0.094) or cancer (*p* = 0.055).

The findings suggest that individuals with SIs exhibit significant differences in various health parameters compared to those without SIs. Individuals with SIs demonstrated notably reduced ADL capacity (*p* < 0.001). Advanced age, male sex, former smoking status, brain damage, intellectual disability, hearing problems, history of stroke, memory-related diseases, hypertension, and dyslipidemia were all significantly associated with an increased risk of SIs (*p* < 0.05). These findings underscore the importance of considering multiple health factors related to SIs in clinical assessments and interventions.

### 3.2 The correlation between covariates and speech impediments

Variables within the demographic characteristics that were not statistically significant, such as residence, education, drinking status, cancer, and diabetes, were excluded. Subsequently, Pearson or Spearman correlation analyses were employed to explore the associations between SIs and various independent and covariates (see Table 3). The results of the correlation analysis revealed a significant negative correlation between ADL (Barthel Index) and SIs (*r* = −0.205, *p* < 0.001), suggesting a marked decline in daily living abilities among individuals with SIs.

**Table 3:** Correlation between covariates and speech impediments.

Variables	Speech impediments	
	r/ρ	*p*-value
Age	0.037	<0.001
Gender	−0.037	<0.001
Smoking status	−0.025	0.012
Brain Damage/Intellectual disability	0.165	<0.001
Hearing Problem	0.092	<0.001
Stroke	0.142	<0.001
Memory-Related Disease	0.081	<0.001
Hypertension	0.033	0.001
Dyslipidemia	0.021	0.033
Activity of daily living (The Barthel Index)	−0.205	<0.001

Speech impediments: 0 = “No,” 1 = “Yes”; Gender: 1 = “Male,” 2 = “Female”; Smoking status: 1 = “Still Smoke,” 2 = “Used to Smoke,” 3 = “Never Smoked”; Brain Damage/Intellectual disability: 0 = “No,” 1 = “Yes”; Hearing Problem/Stroke/Memory-Related Disease/Hypertension/Dyslipidemia: 0 = “No,” 1 = “Yes”.

Furthermore, age was found to be significantly positively correlated with SIs (*r* = 0.037, *p* < 0.001), indicating that the risk of developing SIs may increase with advancing age. A negative correlation was observed between gender and SIs (*ρ* = −0.037, *p* < 0.001), suggesting a higher risk of SIs in males compared to females. Smoking status showed a slight negative correlation with SIs (*ρ* = −0.025, *p* = 0.012), which might imply a potentially protective effect of smoking history against SIs to some extent.

Brain damage/intellectual disability was strongly positively correlated with SIs (*ρ* = 0.165, *p* < 0.001), indicating that these conditions significantly increase the risk of SIs. Hearing problems (ρ = 0.092, *p* < 0.001), a history of stroke (ρ = 0.142, *p* < 0.001), and memory-related diseases (ρ = 0.081, *p* < 0.001) were also significantly positively correlated with SIs, suggesting that these conditions contribute to an elevated risk of SIs. Regarding chronic conditions, both hypertension (ρ = 0.033, *p* = 0.001) and dyslipidemia (ρ = 0.021, *p* = 0.033) were significantly positively associated with the incidence of SIs, indicating that these conditions may increase the risk of SIs. These findings underscore the close association between multiple health conditions and the occurrence of SIs, providing critical insights for further research and the development of intervention strategies.

### 3.3 Associations between ADL and speech impediments

To comprehensively understand the association between SIs and ADL, while accounting for potential confounding factors, we developed three regression models (Model 1, Model 2, Model 3). These models systematically explored the relationships between the independent variables and covariates (such as ADL, age, gender, smoking status, brain damage/intellectual disability, etc.) and the dependent variable (speech impediments).

In Model 1, the analysis included three variables: ADL, age, and gender (R = 0.211, R^2^ = 0.045, *F* = 158.131, *p* < 0.001). The results demonstrated a significant negative correlation between ADL and SIs (B = −0.001, *β* = −0.215, *t* = −21.151, 95% CI = −0.001 to −0.001, *p* = 0.000), suggesting that lower ADL scores are associated with an increased risk of SIs.

In Model 2, we further included smoking status as a variable (R = 0.212, R^2^ = 0.045, *F* = 95.467, *p* < 0.001). The findings indicated that the significant negative correlation between ADL and SIs persisted (B = −0.001, *β* = −0.213, *t* = −20.916, 95% CI = −0.001 to −0.001, *p* = 0.000), suggesting that the inclusion of smoking status did not alter the relationship between ADL and SIs.

Model 3, as the final iteration in this study, incorporated variables related to underlying medical conditions (R = 0.271, R^2^ = 0.073, *F* = 72.907, *p* < 0.001), including brain damage/intellectual disability, hearing problems, history of stroke, memory-related diseases, hypertension, and dyslipidemia. These variables were associated with SIs and could potentially increase susceptibility to SIs. However, even with the inclusion of these medical condition variables, the significant negative correlation between ADL and SIs remained (B = −0.001, *β* = −0.168, *t* = −16.16, 95% CI = −0.001 to −0.001, *p* = 0.000), further confirming the inverse relationship between ADL and SIs.

All three models demonstrated statistically significant associations between the independent variables, covariates, and the dependent variable (*p* < 0.001). The R^2^ value increased progressively from 0.045 in Model 1 to 0.073 in Model 3, indicating an enhancement in the explanatory power of the models. The iterative analysis across these three models consistently highlighted a significant association between decreased ADL and increased susceptibility to SIs, underscoring the robustness of this relationship (Table 4).

**Table 4:** Associations between ADL and speech impediments in middle-aged and older adult Chinese.

Model	R	R square	F	*p*-value	Variables		B	β	*t*	95%CI		*p*-value	VIF
Model 1	0.211	0.045	158.131	<0.001	ADL		−0.001	−0.215	−21.151	−0.001	−0.001	0.000	1.091
					Age		0.000	−0.027	−2.676	0.000	0.000	0.007	1.096
					Gender	Male	0.011	0.049	5.053	0.006	0.015	0.000	1.012
						Female(Ref.)							
Model 2	0.212	0.045	95.467	<0.001	ADL		−0.001	−0.213	−20.916	−0.001	−0.001	0.000	1.1
					Age		0.000	−0.029	−2.807	0.000	0.000	0.005	1.104
					Gender	Male	0.010	0.046	3.260	0.004	0.016	0.001	2.078
						Female(Ref.)							
					Smoking status	Still Smoke	−0.001	−0.005	−0.364	−0.008	0.005	0.716	1.827
						Used to Smoke	0.005	0.015	1.234	−0.003	0.012	0.217	1.661
						Never Smoked(Ref.)							
Model 3	0.271	0.073	72.907	<0.001	ADL		−0.001	−0.168	−16.160	−0.001	−0.001	0.000	1.183
					Age		0.000	−0.033	−3.291	−0.001	0.000	0.001	1.117
					Gender	Male	0.008	0.039	2.800	0.002	0.014	0.005	2.082
						Female(Ref.)							
					Smoking status	Still Smoke	0.000	0.001	0.067	−0.006	0.007	0.946	1.83
						Used to Smoke	0.003	0.011	0.870	−0.004	0.010	0.384	1.662
						Never Smoked(Ref.)							
					Brain Damage/Intellectual disability	Yes	0.053	0.108	10.771	0.044	0.063	0.000	1.097
						No(Ref.)							
					Hearing Problem	Yes	0.024	0.062	6.364	0.016	0.031	0.000	1.028
						No(Ref.)							
					Stroke	Yes	0.037	0.091	9.260	0.029	0.044	0.000	1.062
						No(Ref.)							
					Memory-Related Disease	Yes	0.010	0.019	1.873	0.000	0.021	0.061	1.073
						No(Ref.)							
					Hypertension	Yes	0.003	0.011	1.080	−0.002	0.008	0.280	1.034
						No(Ref.)							
					Dyslipidemia	Yes	−0.001	−0.002	−0.256	−0.007	0.005	0.798	1.035
						No(Ref.)							

Considering age as a significant confounding factor, we stratified the study population into two distinct groups to mitigate the potential impact of age on the dependent variable: the middle-aged group (≥45 and < 60 years; Table 5) and the older-adult group (≥60 years; Table 6). Analysis of models stratified by age groups revealed a significant negative correlation between ADL and SIs in both the middle-aged group (B = −0.001, *β* = −0.129, *t* = −7.39, 95% CI = −0.001 to −0.001, *p* = 0.000) and the older adult group (B = −0.001, *β* = −0.175, *t* = −13.888, 95% CI = −0.001 to −0.001, *p* = 0.000). Furthermore, this negative correlation persisted across all iterations of Model 1, Model 2, and Model 3, irrespective of the covariate adjustments made. These findings robustly reinforce our conclusion of a negative association between ADL and SIs.

**Table 5:** Associations between ADL and SIs in middle-aged group.

Model	R	R square	F	*p*-value	Variables		B	β	*t*	95%CI		*p*-value	VIF
Model 1	0.194	0.037	42.589	<0.001	ADL		−0.001	−0.193	−11.196	−0.001	−0.001	0.000	1.016
					Age		−0.001	−0.032	−1.859	−0.002	0.000	0.063	1.01
					Gender	Male	0.007	0.041	2.362	0.001	0.013	0.018	1.012
						Female(Ref.)							
Model 2	0.195	0.038	26.061	<0.001	ADL		−0.001	−0.191	−11.034	−0.001	−0.001	0.000	1.022
					Age		−0.001	−0.033	−1.925	−0.002	0.000	0.054	1.012
					Gender	Male	0.005	0.026	1.017	−0.004	0.014	0.309	2.200
						Female(Ref.)							
					Smoking status	Still Smoke	0.001	0.006	0.248	−0.009	0.011	0.804	1.954
						Used to Smoke	0.009	0.031	1.489	−0.003	0.021	0.137	1.520
						Never Smoked(Ref.)							
Model 3	0.309	0.096	31.469	<0.001	ADL		−0.001	−0.129	−7.390	−0.001	−0.001	0.000	1.105
					Age		−0.001	−0.032	−1.940	−0.002	0.000	0.052	1.013
					Gender	Male	0.004	0.021	0.831	−0.005	0.012	0.406	2.216
						Female(Ref.)							
					Smoking status	Still Smoke	0.002	0.007	0.318	−0.008	0.011	0.751	1.960
						Used to Smoke	0.004	0.014	0.673	−0.008	0.016	0.501	1.527
						Never Smoked(Ref.)							
					Brain Damage/Intellectual disability	Yes	0.056	0.127	7.176	0.041	0.072	0.000	1.130
						No(Ref.)							
					Hearing Problem	Yes	0.037	0.103	6.085	0.025	0.049	0.000	1.035
						No(Ref.)							
					Stroke	Yes	0.030	0.071	4.086	0.016	0.045	0.000	1.095
						No(Ref.)							
					Memory-Related Disease	Yes	0.080	0.109	6.345	0.055	0.105	0.000	1.074
						No(Ref.)							
					Hypertension	Yes	−0.003	−0.012	−0.707	−0.011	0.005	0.480	1.041
						No(Ref.)							
					Dyslipidemia	Yes	−0.001	−0.002	−0.144	−0.009	0.008	0.886	1.036
						No(Ref.)							

**Table 6:** Associations between ADL and SIs in older-adult group.

Model	R	R square	F	*p*-value	Variables		B	β	*t*	95%CI		*p*-value	VIF
Model 1	0.215	0.046	110.766	<0.001	ADL		−0.001	−0.216	−17.646	−0.001	−0.001	0.000	1.073
					Age		0.000	−0.019	−1.596	−0.001	0.000	0.111	1.071
					Gender	Male	0.012	0.053	4.437	0.007	0.017	0.000	1.005
						Female(Ref.)							
Model 2	0.216	0.046	66.717	<0.001	ADL		−0.001	−0.215	−17.472	−0.001	−0.001	0.000	1.082
					Age		0.000	−0.020	−1.668	−0.001	0.000	0.095	1.076
					Gender	Male	0.012	0.052	3.099	0.004	0.019	0.002	2.014
						Female(Ref.)							
					Smoking status	Still Smoke	−0.002	−0.007	−0.473	−0.010	0.006	0.636	1.781
						Used to Smoke	0.003	0.01	0.638	−0.006	0.012	0.523	1.691
						Never Smoked(Ref.)							
Model 3	0.266	0.071	47.185	<0.001	ADL		−0.001	−0.175	−13.888	−0.001	−0.001	0.000	1.164
					Age		0.000	−0.019	−1.565	−0.001	0.000	0.118	1.087
					Gender	Male	0.010	0.045	2.720	0.003	0.018	0.007	2.018
						Female(Ref.)							
					Smoking status	Still Smoke	0.000	−0.001	−0.065	−0.009	0.008	0.949	1.786
						Used to Smoke	0.002	0.008	0.532	−0.006	0.011	0.594	1.692
						Never Smoked(Ref.)							
					Brain Damage/Intellectual disability	Yes	0.051	0.100	8.249	0.039	0.063	0.000	1.09
						No(Ref.)							
					Hearing Problem	Yes	0.019	0.048	4.054	0.010	0.028	0.000	1.024
						No(Ref.)							
					Stroke	Yes	0.037	0.094	7.860	0.028	0.046	0.000	1.051
						No(Ref.)							
					Memory-Related Disease	Yes	0.001	0.001	0.095	−0.012	0.013	0.924	1.068
						No(Ref.)							
					Hypertension	Yes	0.005	0.018	1.521	−0.002	0.012	0.128	1.031
						No(Ref.)							
					Dyslipidemia	Yes	0.000	−0.001	−0.118	−0.008	0.007	0.906	1.038
						No(Ref.)							

Certain comorbidities are known to influence the occurrence of SIs. To minimize the confounding effects of these comorbidities, we further stratified the study sample into two cohorts: a comorbidity group (Table 7), comprising individuals diagnosed with one or more conditions such as brain damage or intellectual disability, hearing problems, stroke, memory-related diseases, hypertension, or dyslipidemia, and a non-comorbidity group (Table 8), consisting of individuals without any of these conditions. Subsequent analyses of these two cohorts revealed that in both the comorbidity group (B = −0.002, *β* = −0.24, *t* = −14.423, 95% CI = −0.002 to −0.001, *p* = 0.000) and the non-comorbidity group (B = 0.000, *β* = −0.061, *t* = −4.665, 95% CI = 0.000 to 0.000, *p* = 0.000), there was a statistically significant negative correlation between ADL and SIs, although the association was relatively weaker in the non-comorbidity group. These findings suggest that the decline in ADL is more strongly associated with increased SIs in individuals with comorbid conditions, potentially due to an increased vulnerability conferred by these conditions, which amplifies the impact of reduced ADL on speech function. Conversely, in the absence of comorbidities, this negative correlation, although weaker, remains significant. After adjusting for the confounding effects of comorbidities, ADL continues to show a negative correlation with SIs.

**Table 7:** Associations between ADL and SIs in the comorbidity group.

Model	R	R square	F	*p*-value	Variables	B		β	*t*	95%CI		*p*-value	VIF
Model 1	0.286	0.082	114.996	<0.001	ADL		−0.002	−0.292	−18.146	−0.002	−0.002	0.000	1.092
					Age		−0.001	−0.041	−2.547	−0.001	0.000	0.011	1.094
					Gender	Male	0.017	0.058	3.772	0.008	0.026	0.000	1.007
						Female(Ref.)							
Model 2	0.286	0.082	69.412	<0.001	ADL		−0.002	−0.289	−17.888	−0.002	−0.002	0.000	1.105
					Age		−0.001	−0.042	−2.628	−0.001	0.000	0.009	1.099
					Gender	Male	0.016	0.055	2.512	0.004	0.029	0.012	2.032
						Female(Ref.)							
					Smoking status	Still Smoke	−0.003	−0.009	−0.441	−0.018	0.011	0.659	1.794
						Used to Smoke	0.007	0.019	0.942	−0.008	0.022	0.346	1.664
						Never Smoked(Ref.)							
Model 3	0.337	0.114	45.228	<0.001	ADL		−0.002	−0.240	−14.423	−0.002	−0.001	0.000	1.214
					Age		−0.001	−0.044	−2.748	−0.001	0.000	0.006	1.135
					Gender	Male	0.013	0.044	2.020	0.000	0.025	0.044	2.042
						Female(Ref.)							
					Smoking status	Still Smoke	0.000	0.000	0.009	−0.014	0.014	0.993	1.803
						Used to Smoke	0.005	0.013	0.660	−0.010	0.020	0.509	1.668
						Never Smoked(Ref.)							
					Brain Damage/Intellectual disability	Yes	0.055	0.123	7.793	0.041	0.069	0.000	1.097
						No(Ref.)							
					Hearing Problem	Yes	0.040	0.110	6.709	0.028	0.051	0.000	1.182
						No(Ref.)							
					Stroke	Yes	0.045	0.123	7.450	0.033	0.057	0.000	1.185
						No(Ref.)							
					Memory-Related Disease	Yes	0.015	0.029	1.845	−0.001	0.03	0.065	1.104
						No(Ref.)							
					Hypertension	Yes	0.022	0.074	4.362	0.012	0.032	0.000	1.268
						No(Ref.)							
					Dyslipidemia	Yes	0.016	0.053	3.155	0.006	0.026	0.002	1.213
						No(Ref.)							

**Table 8:** Associations between ADL and SIs in the non-comorbidity group.

Model	R	R square	F	*p*-value	Variables		B	β	*t*	95%CI		*p*-value	VIF
Model 1	0.069	0.005	9.933	<0.001	ADL		0.000	−0.062	−4.718	0.000	0.000	0.000	1.083
					Age		0.000	−0.005	−0.385	0.000	0.000	0.700	1.09
					Gender	Male	0.005	0.037	2.872	0.002	0.008	0.004	1.017
						Female(Ref.)							
Model 2	0.070	0.005	6.071	<0.001	ADL		0.000	−0.061	−4.665	0.000	0.000	0.000	1.088
					Age		0.000	−0.006	−0.419	0.000	0.000	0.675	1.099
					Gender	Male	0.004	0.027	1.472	−0.001	0.008	0.141	2.112
						Female(Ref.)							
					Smoking status	Still Smoke	0.002	0.01	0.574	−0.004	0.007	0.566	1.85
						Used to Smoke	0.002	0.012	0.711	−0.004	0.008	0.477	1.656
						Never Smoked(Ref.)							

### 3.4 Machine learning algorithm confirm the link between ADL and speech impediments

**Figure 1:**
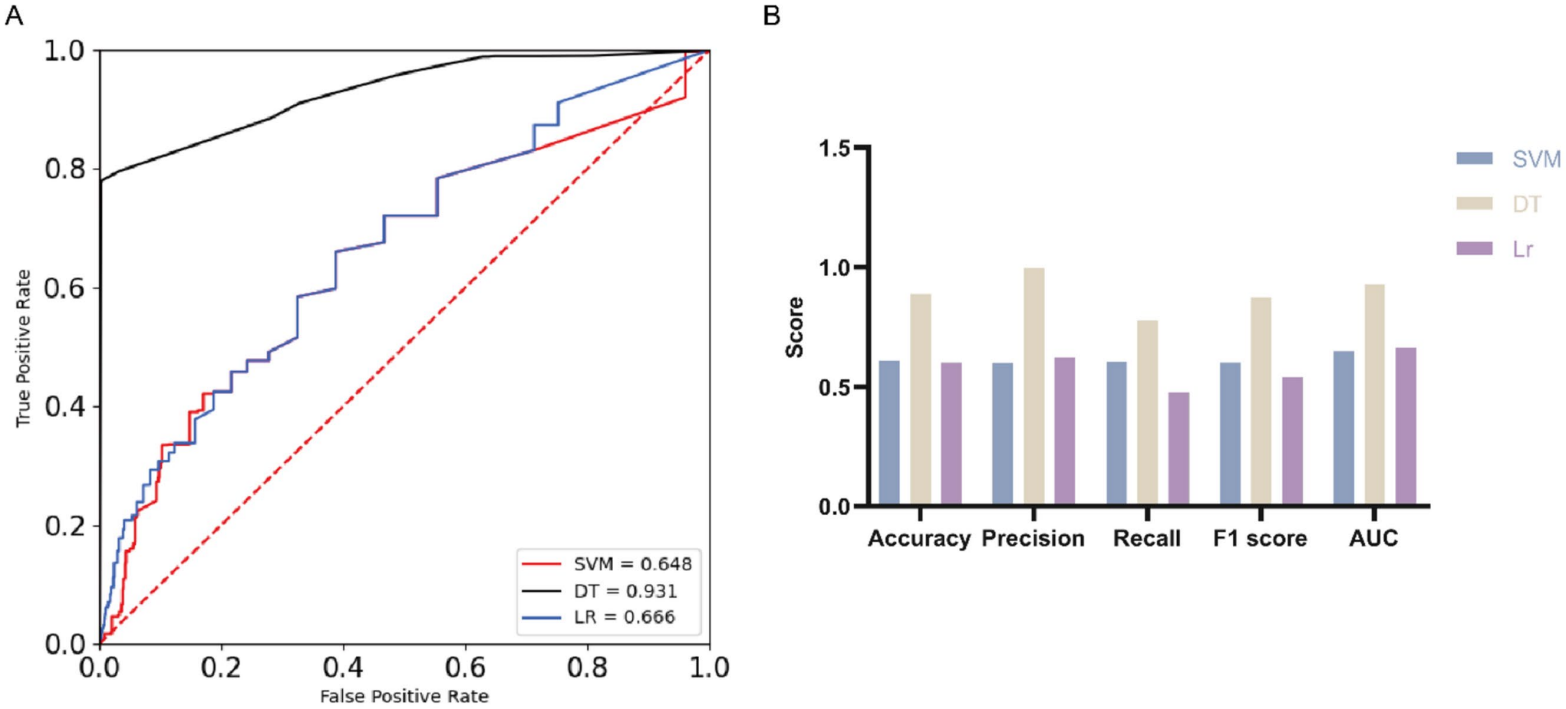
ROC curves and evaluation indicators. **(A)** ROC curves with AUC for the three machine learning algorithms. **(B)** Comparison of three machine learning algorithms.

Based on the statistical analysis, we observed a significant inverse correlation between ADL and SIs, indicating that a decline in ADL is associated with an increased susceptibility to SIs. To further validate this observation, we employed three machine learning algorithms—SVM, DT, and LR—to perform additional analyses (Table 9; Figure 1). The results demonstrated that all three models had an AUC greater than 0.6, suggesting a reasonable degree of predictive accuracy across the models.

**Table 9:** Evaluation of the three machine learning algorithms.

Model	Accuracy	Precision	Recall	F1 score	AUC
SVM	0.609	0.601	0.605	0.603	0.648
DT	0.890	0.997	0.779	0.875	0.931
Lr	0.602	0.624	0.478	0.541	0.666

Among these, the DT model exhibited the best performance, with an accuracy of 0.890, a precision of 0.997, a recall of 0.779, an F1 score of 0.875, and an AUC of 0.931, indicating superior performance across all metrics. In contrast, the SVM showed moderate performance, with an accuracy of 0.609, a precision of 0.601, a recall of 0.605, an F1 score of 0.603, and an AUC of 0.648. The LR model demonstrated relatively weaker classification ability, with an accuracy of 0.602, a precision of 0.624, a recall of 0.478, an F1 score of 0.541, and an AUC of 0.666.

**Figure 2:**
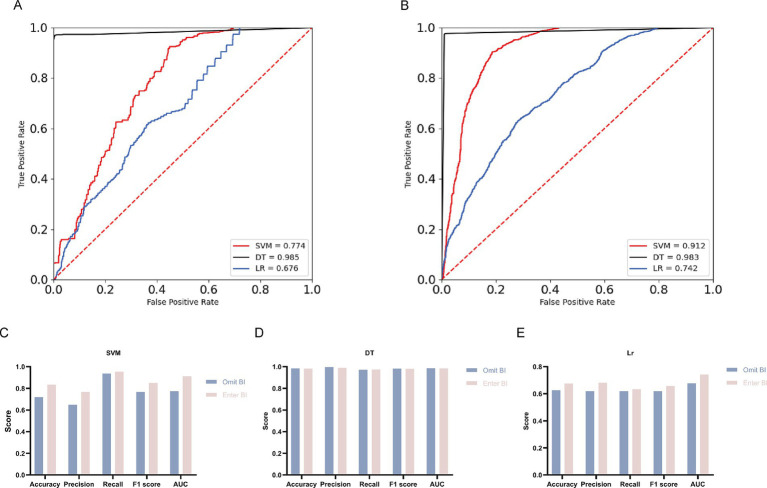
Comparative analysis of machine learning algorithm performance on two distinct datasets. **(A)** ROC curves and AUC values for the group excluding the Barthel Index (Omit BI Group). **(B)** ROC curves and AUC values for the group including the Barthel Index (Enter BI Group). **(C)** Comparison of SVM model performance between the Omit BI and Enter BI groups. **(D)** Comparison of DT model performance between the Omit BI and Enter BI groups. **(E)** Comparison of LR model performance between the Omit BI and Enter BI groups.

To mitigate the impact of potential confounding factors and to strengthen the credibility of the observed association between ADL and SIs, we examined the influence of incorporating the BI on the predictive performance of three machine learning algorithms: SVM, DT, and LR (Table 10; Figure 2). The results indicate that the inclusion of BI improved the predictive capabilities of all models.

**Table 10:** Overall evaluation of three machine learning algorithms for two datasets.

Model	Accuracy		Precision		Recall		F1 score		AUC	
	Omit BI	Enter BI	Omit BI	Enter BI	Omit BI	Enter BI	Omit BI	Enter BI	Omit BI	Enter BI
SVM	0.719	0.834	0.648	0.766	0.936	0.953	0.766	0.849	0.774	0.912
DT	0.983	0.982	0.995	0.989	0.970	0.973	0.982	0.981	0.985	0.983
Lr	0.626	0.675	0.619	0.681	0.619	0.634	0.619	0.657	0.676	0.742

For the SVM model, the incorporation of BI resulted in a notable enhancement of predictive performance. Specifically, accuracy increased from 0.719 to 0.834, precision from 0.648 to 0.766, recall from 0.936 to 0.953, F1 score from 0.766 to 0.849, and AUC from 0.774 to 0.912. These improvements suggest that the inclusion of BI significantly enhanced the SVM model’s ability to distinguish between individuals with and without SIs. In the case of the DT model, although the differences in performance before and after the inclusion of BI were minimal, the model consistently exhibited high predictive capability under both conditions. Following the inclusion of BI, the accuracy slightly decreased from 0.983 to 0.982, precision decreased from 0.995 to 0.989, recall increased from 0.970 to 0.973, F1 score decreased from 0.982 to 0.981, and AUC decreased from 0.985 to 0.983. Despite these minor changes, the overall performance remained at a high level, demonstrating the stability and robustness of the DT model. The LR model also showed improved performance upon the inclusion of BI. Accuracy increased from 0.626 to 0.675, precision from 0.619 to 0.681, recall from 0.619 to 0.634, F1 score from 0.619 to 0.657, and AUC from 0.676 to 0.742. Although the overall performance of the LR model was not as strong as that of the SVM and DT models, its classification effectiveness was significantly improved with the addition of BI.

These findings suggest that incorporating the BI enhances the predictive accuracy of all models, particularly in the SVM and LR models. The inclusion of BI markedly improved the models’ ability to identify the risk of SIs, reinforcing the strong association between ADL and SIs observed in our statistical analyses. This supports the validity of our initial findings.

## 4 Discussion

As the global population ages, the prevalence of speech impediments significantly affects the quality of life among middle-aged and older adult individuals (30). Given the complex etiology of SIs, early detection and intervention are crucial.

In this study, we first performed a demographic analysis of the study population and observed a significant difference in the mean BI scores between the cohorts with and without SIs. The cohort with SIs exhibited substantially lower BI scores compared to those without. Further correlation analysis, utilizing Pearson or Spearman correlation tests, revealed a significant negative correlation between BI scores and SIs. Even after adjusting for potential confounders such as age, gender, lifestyle factors, and comorbidities, ADL remained significantly negatively correlated with SIs. This finding was further corroborated by machine learning algorithms. In experiments predicting SIs using BI, all three machine learning models achieved AUC values exceeding 0.6, with the DT model achieving an AUC as high as 0.931, indicating diagnostic value across models. Comparative experiments—both with all variables and excluding BI—demonstrated that including BI consistently enhanced the predictive performance of the models. This reinforces our findings on the association between ADL and SIs.

We also examined other covariates in our study. In the analysis of demographic characteristics and correlations, variables such as age, gender, smoking status, brain damage or intellectual disability, hearing problems, stroke, memory-related diseases, hypertension, and dyslipidemia showed significant differences and correlations between the two cohorts (with and without speech impediments). After conducting stratified multiple linear regression analysis with three iterations across different models, controlling for these variables, age, gender, brain damage/intellectual disability, hearing problems, and stroke remained significantly associated with SIs. Specifically, older individuals were more likely to experience SIs, and those with a history of brain damage, intellectual disability, hearing problems, or stroke were at greater risk. This is consistent with previous research findings (31–34). The finding that men are more prone to SIs than women lacks a definitive conclusion in the existing literature; One possible explanation could be the higher risk of stroke and other cerebrovascular events in middle-aged and older adult men compared to women (35). Cerebrovascular diseases disrupt neural networks critical for speech production by impairing regions such as Broca’s and Wernicke’s areas, along with cortico-subcortical pathways (36–38). These impairments compromise phonological and semantic processes, including word retrieval and sentence comprehension. Recent studies highlight that damage to these areas disrupts early semantic memory binding and syntactic integration, leading to significant SIs. Further exploration using multimodal approaches like MRI and EEG could provide deeper insights into the underlying neurobiological mechanisms (39, 40). Our study did not include data on Parkinson’s disease, a condition frequently associated with speech difficulties such as reduced vocal volume or altered pitch, which has a higher incidence in men (41, 42). Furthermore, estrogen, which has neuroprotective effects, promoting neuron survival and function, is higher in women pre-menopause and might still offer some protection against language decline even post-menopause. In contrast, declining testosterone levels in middle-aged and older adult men could adversely affect brain function, particularly areas related to speech (43, 44).

Our findings demonstrate a significant negative correlation between ADL and SIs, which remains robust even after adjusting for other covariates, a conclusion further validated by machine learning models. This underscores the importance of promoting ADL training among older adults, particularly men and those with underlying conditions. In community-based elder care and family caregiving, enhancing ADL capabilities among older adults is crucial for preventing Sis (45) and maintaining quality of life. For those already experiencing SIs, improving ADL may aid in recovery and reduce the burden on individuals, families, and society (46).

Despite the observed significant negative correlation between SIs and ADL, as well as the validation of these findings through machine learning algorithms, this study has several limitations. Firstly, as a cross-sectional analysis, we did not investigate the causes of SIs or track subsequent changes in the participants, making it impossible to establish a causal relationship between ADL and SIs. It is also plausible that reduced ADL is a consequence of SIs. Thus, we can only conclude a significant negative correlation between ADL and SIs. Secondly, the multitude of factors contributing to SIs was not exhaustively analyzed in this study, which may introduce some bias into the results. Finally, ADL was assessed using the BI, which was derived from corresponding items in the CHARLS dataset based on ADL questionnaires. SIs were defined using recorded results from CHARLS data. These data sources are self-reported; while self-reports may differ from clinical assessments in certain respects, some studies support the reliability of self-reported data. Therefore, the findings of this study remain of significant importance (47–50).

## 5 Conclusion

This study employed a range of statistical methods to identify a negative correlation between ADL and SIs. This relationship was further validated using machine learning algorithms, confirming that impaired ADL is associated with an increased incidence of SIs.

## Data Availability

The original contributions presented in the study are included in the article/supplementary material, further inquiries can be directed to the corresponding author.

## Footnotes

^1^ http://charls.pku.edu.cn
